# Secreted Phospholipases A_2_ - not just Enzymes: Revisited

**DOI:** 10.7150/ijbs.68093

**Published:** 2022-01-01

**Authors:** Adrijan Ivanušec, Jernej Šribar, Igor Križaj

**Affiliations:** 1 Department of Molecular and Biomedical Sciences, Jožef Stefan Institute, Jamova 39, 1000 Ljubljana, Slovenia.; 2 Faculty of Medicine, University of Ljubljana, Vrazov trg 2, 1000 Ljubljana, Slovenia.

**Keywords:** Secreted phospholipase A_2_, binding protein, promiscuity, cell transport, signalling, phospholipase activity regulation

## Abstract

Secreted phospholipases A_2_ (sPLA_2_s) participate in a very broad spectrum of biological processes through their enzymatic activity and as ligands for membrane and soluble receptors. The physiological roles of sPLA_2_s as enzymes have been very well described, while their functions as ligands are still poorly known. Since the last overview of sPLA_2_-binding proteins (sPLA_2_-BPs) 10 years ago, several important discoveries have occurred in this area. New and more sensitive analytical tools have enabled the discovery of additional sPLA_2_-BPs, which are presented and critically discussed here. The structural diversity of sPLA_2_-BPs reveals sPLA_2_s as very promiscuous proteins, and we offer some structural explanations for this nature that makes these proteins evolutionarily highly advantageous. Three areas of physiological engagement of sPLA_2_-BPs have appeared most clearly: cellular transport and signalling, and regulation of the enzymatic activity of sPLA_2_s. Due to the multifunctionality of sPLA_2_s, they appear to be exceptional pharmacological targets. We reveal the potential to exploit interactions of sPLA_2_s with other proteins in medical terms, for the development of original diagnostic and therapeutic procedures. We conclude this survey by suggesting the priority questions that need to be answered.

## Introduction

Secreted phospholipases A_2_ (sPLA_2_s) (EC 3.1.1.4) are a structurally related group of low-molecular-mass enzymes (14-18 kDa) that catalyse the hydrolysis of glycerophospholipids (phospholipids hereafter) at their *sn*-2 position, to produce lysophospholipids and free fatty acids. sPLA_2_s contain 6 to 8 disulphide bonds, a highly conserved His/Asp catalytic dyad, and a Ca^2+^-binding loop 1. Several sPLA_2_ isoforms have been described in mammals. Depending on their structural characteristics, the mammalian sPLA_2_s are divided into 11 groups (G): IB, IIA, IIC, IID, IIE, IIF, III, V, X, XIIA and XIIB 2. Snake venom sPLA_2_s are orthologous to mammalian GIIA, GIIB or GIIE sPLA_2_s, or they belong to the unique GIA sPLA_2_s 3. sPLA_2_s from bee and lizard venoms are homologous to mammalian GIII sPLA_2_s. Finally, the further GIX sPLA_2_s are found in venom of marine snails, and GXIA and GXIB sPLA_2_s are plant proteins.

## The importance of sPLA_2_s as enzymes

The enzymatic activity of sPLA_2_s define their participation in a very broad spectrum of biological processes. Generally, sPLA_2_s are secreted from cells and require micromolar to millimolar Ca^2+^ to be catalytically competent. They predominantly target phospholipids in the extracellular space, although they also act intracellularly.

sPLA_2_s show distinct substrate specificities in terms of the phospholipid polar headgroups and the fatty acids esterified at the *sn-*2 position of the glycerol backbone. For instance, GIII, GV and GX sPLA_2_s efficiently hydrolyse phosphatidylcholine, while GIIA sPLA_2_ has higher activity against negatively charged phospholipid substrates, and in particular, phosphatidylserine, phosphatidylglycerol and phosphatidylethanolamine. For their *sn*-2 fatty acid tail specificities, GIB, GIIA and GIIE sPLA_2_s are promiscuous. GV sPLA_2_ preferentially targets *sn*-2 fatty acyls with low number of double bonds, such as oleyl, while GIID, GIIF, GIII and GX sPLA_2_s target polyunsaturated fatty acyls, such as arachidonyl.

sPLA_2_ isoforms have unique tissue and cellular distributions, and therefore it is evident that individual sPLA_2_s have distinct enzyme-activity-related biological functions. These include the generation of a variety of lipid mediators, along with membrane remodelling, modification of extracellular non-cellular phospholipid components of pulmonary surfactant, microparticles and lipoproteins, and degradation of microbial membranes and dietary phospholipids. The pathophysiological aspects of sPLA_2_s as enzymes have been comprehensively reviewed recently (e.g., 2,4), and therefore we will not focus on these here. Instead, our attention here is focussed on sPLA_2_s acting as ligands, where fewer of the details have been described.

The existence of sPLA_2_s without enzymatic activity but with pharmacological activity was an early indication that sPLA_2_s can participate in physiological settings not just as enzymes, but also as ligands for membrane and soluble receptors. Indeed, in 1982, the first specific sPLA_2_-binding protein (sPLA_2_-BP) was discovered 5. Since then, the number of newly characterized sPLA_2_-BPs has expanded considerably (Table 1), and due to the development of more sensitive analytical methodologies, this process continues. Several reviews on sPLA_2_-BPs have been published over the years 6-9, although the most recent is 10 years old 9. Therefore, now is an appropriate time to survey the advances in this important area of research, and to critically discuss new insights and suggest research directions.

## sPLA_2_s bind very structurally diverse proteins

There are two types of sPLA_2_-BPs: integral membrane proteins and soluble proteins 9. The former belong to one of the following protein families: (1) muscle-type sPLA_2_ receptors (M-type sPLA_2_Rs); (2) heparan sulphate proteoglycans (HSPGs); (3) integrins; (4) vascular endothelial growth factor receptors (VEGFRs); and (5) ion channels. Two new integral membrane sPLA_2_-BPs have been described recently, a G-protein-coupled receptor (GPCR), and cytochrome c oxidase (CCOX) 35, 37. CCOX is the only known intracellular membrane sPLA_2_-BP, as all of the other integral membrane sPLA_2_-BPs are located in the plasma membrane. On the other hand, soluble sPLA_2_-BPs are more frequently found inside cells, in the endoplasmic reticulum (ER), cytosol or nucleus, as well as in the extracellular space. The ER-resident sPLA_2_-BPs, disulphide isomerase (PDI), taipoxin-associated 49-kDa Ca^2+^-binding protein (TCBP-49) and crocalbin, and the cytosolic calmodulin (CaM) and 14-3-3 proteins, have been known for some time. Two new soluble sPLA_2_-BPs were only described more recently: vimentin 26 and nucleolin 27. Vimentin is an intermediate filament protein, as a constituent of the cytoskeleton, while nucleolin is a nucleolar protein; however, both of these proteins are also found on the cell surface, which is where they are most likely to interact with sPLA_2_s. Extracellular sPLA_2_-BPs include pentraxins, α-, β- and γ-type sPLA_2_ inhibitors (PLIs), blood coagulation factors, pulmonary surfactant protein A (SP-A), metalloproteinase inhibitor DM64, and a serine protease inhibitor (C1 inhibitor protein). The main characteristics of the sPLA_2_-BPs are shown in Table 1.

## Structural aspects of sPLA_2_ protein-binding-promiscuity

sPLA_2_-BPs are structurally so diverse that attempts to define their common sPLA_2_-binding attributes have remained unsuccessful. To solve this problem, it would be most helpful to determine the three-dimensional structures of complexes between sPLA_2_s and the sPLA_2_-BPs, and thus to analyse their interaction areas. However, contemporary methodologies are still not optimal for this kind of approach.

**Figure 1:**
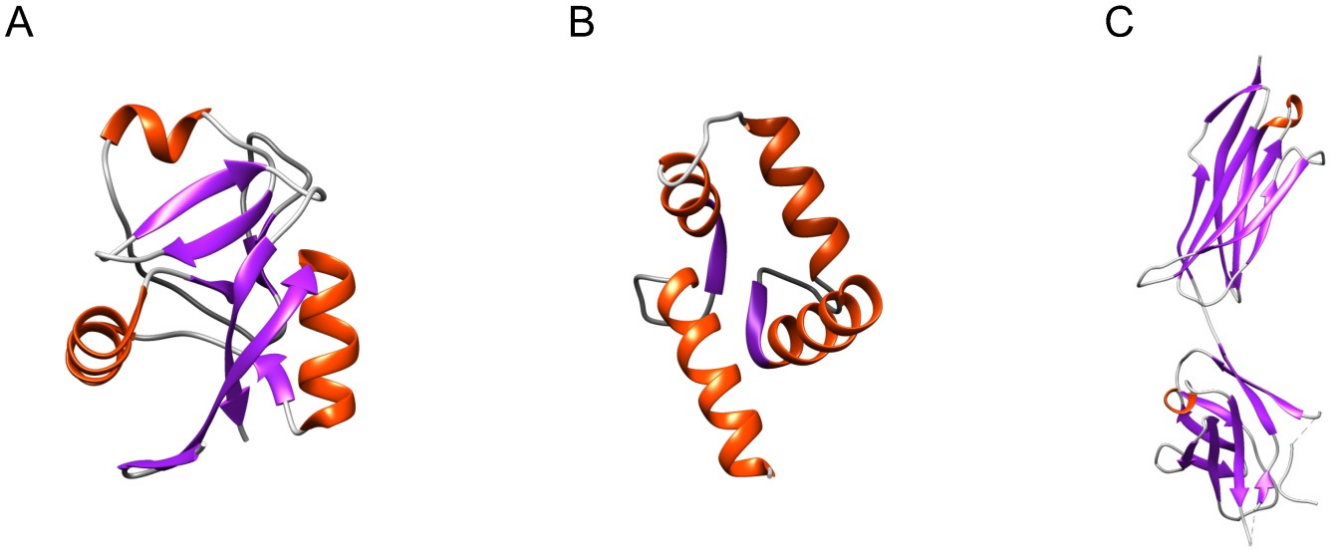
** Distinct structural elements present in more than one type of sPLA_2_-BP.** (**A**) CRD (PDB ID: 6JLI) and the CRD-like fold are structural elements found in M-type sPLA_2_Rs, SP-A and α-PLIs. (**B**) The EF-hand Ca^2+^-binding motif (PDB ID: 1CLL) is found in CaM, crocalbin and TCBP-49, which all bind sPLA_2_s. (**C**) The Ig-like domain (PDB ID: 2X1X) is found in VEGFRs and DM64. To date, only the CRD has been experimentally demonstrated to be a sPLA_2_-binding structure. Red, α-helices; violet, β-sheets; grey, loops. The Figure was prepared using UCSF Chimera v1.15.

The focus has thus mainly been on well-defined structural elements that are present in more than one type of sPLA_2_-BP, such as the C-type carbohydrate recognition domain (CRD) and CRD-like folds (in M-type sPLA_2_Rs, SP-A, α-PLIs), the EF-hand Ca^2+^-binding motif (in CaM, crocalbin, TCBP-49), and the immunoglobulin-like (Ig-like) domain (in VEGFRs, DM64) (Figure 1).

Only the CRD fold (Figure 1A) has been confirmed as an sPLA_2_-binding structure 10. Using radio-iodinated snake venom GIA sPLA_2_ (^125^I-OS_1_), it was established that one of the eight CRDs in M-type sPLA_2_R (i.e., CRD5) includes the sPLA_2_ binding site. This also revealed that not all of the CRDs can bind sPLA_2_s. M-type sPLA_2_R bound sPLA_2_s only under neutral and basic conditions, which implies that positively charged amino acids have important roles in this interaction. At pH 6 or lower, M-type sPLA_2_R also underwent conformational changes that prevented the binding of sPLA_2_s 41. For the other two CRD-fold-containing sPLA_2_-BPs, as lung SP-A 13 and soluble α-PLIs from sera 12, the sPLA_2_-binding characteristics are not very clear yet. As M-type sPLA_2_R and α-PLIs do not require Ca^2+^ to bind sPLA_2_s 12, 42, while SP-A does require Ca^2+^, this indicates that CRDs bind sPLA_2_s in different ways. To date, Ca^2+^-dependent binding of sPLA_2_s has been reported only in a case of a single CRD-containing sPLA_2_-BP, SP-A, while Ca^2+^-independent binding of sPLA_2_s has been shown for sPLA_2_-BPs with multiple CRDs, where more than one CRD constitutes the sPLA_2_-binding site. For M-type sPLA_2_R, three consecutive CRD repeats (repeats 4, 5, 6) were shown to influence sPLA_2_ binding 10, and consistently, in α-PLIs, the trimeric CRD proteins, the central part, where three CRDs are associated together, has been suggested to provide the sPLA_2_-binding site 43.

There are plenty of other CRD-containing proteins, such as the mannose receptor, Endo180, DEC205 and FcRY. At present, only the human mannose receptor has been demonstrated to interact with bee venom GIII sPLA_2_ 44, while FcRY did not bind any of the sPLA_2_s tested 45.

To recognize sPLA_2_-binding attributes of sPLA_2_-BPs it is important to also consider the characteristics of the sites on sPLA_2_s that interact with sPLA_2_-BPs, as these are complementary to the sPLA_2_-binding sites on sPLA_2_-BPs. It has to be borne in mind, however, that a single sPLA_2_ molecule might harbour multiple protein-binding sites 46, and therefore it is reasonable to consider separately binding sites directed towards particular types of sPLA_2_-BPs, or those sPLA_2_s that induce the same interaction-dependent physiological effects.

The interaction of an sPLA_2_ with a CRD fold is primarily governed by the structure of the interfacial binding surface (IBS) of the sPLA_2_ 47. The IBS is defined as the molecular surface with which sPLA_2_ contacts a phospholipid membrane - its substrate. The IBS is formed by a collar of hydrophobic amino acids around the entrance to the catalytic site of the enzyme, and by mainly positively charged amino acids in more remote positions. The amino acids within or close to the Ca^2+^-binding loop in sPLA_2_s were also shown to be involved in the interactions between sPLA_2_s and CRDs 42; however, not profoundly, consistent with the evidence that the Ca^2+^-binding loop is highly conserved among sPLA_2_s that show considerably different affinities for M-type sPLA_2_R.

sPLA_2_-BPs that contain Ig-like domains, such as VEGFRs and DM64, have been shown to associate with catalytically inactive myotoxic sPLA_2_s. VEGFRs are receptor tyrosine kinases, and they interact with sPLA_2_s through their extracellular region, which is composed of seven Ig-like domains. Although the interaction of myotoxic sPLA_2_s and VEGFRs has not been functionally linked with myotoxic effects yet, the C-terminal region of these sPLA_2_s (known as the 'myotoxic site') was shown to also be responsible for binding to VEGFRs 22, 23. The myotoxic site of the snake venom myotoxic sPLA_2_s has been attributed to amino acids 115-129, which form a single α-helical turn at the C-terminus 48-50. This C-terminal region is firmly attached to the body of the protein by two disulphide bonds, Cys27-Cys126 and Cys50-Cys133, thus constraining its position and orientation. The myotoxicity of sPLA_2_s has been associated with a cluster of positively charged amino acids in this region (i.e., conserved Lys115 and Arg118, and possibly also Lys122 and Lys127), and with hydrophobic amino acids at positions 121 and 125 51, 52.

The sPLA_2_-BP DM64 was originally isolated from marsupial (*Didelphis marsupialis*) blood, and it was shown to inhibit the myotoxic activity of several sPLA_2_s 24. DM64 contains five Ig-like domains. Its myotoxicity-inhibitory activity is most likely due to its binding to the myotoxic site of sPLA_2_s, to thus obstruct their association with the 'myotoxicity receptor', which would be VEGFR. Contrary to what is seen for CRD in M-type sPLA_2_R, binding of sPLA_2_s to DM64 does not inhibit their enzymatic activity. This is in agreement with the involvement of the C-terminal of these sPLA_2_s in the interaction with this Ig-like domain-containing sPLA_2_-BP, rather than the IBS.

Identification of the PDZ-binding domain at the C-terminus of some myotoxic sPLA_2_s 53 suggested that there is another group of sPLA_2_-BPs, as PDZ-domain-containing proteins. In muscle cells, these proteins include, e.g., LDB3 and α-1-syntrophin 54, 55; however, their potential sPLA_2_ binding remains to be tested.

**Figure 2:**
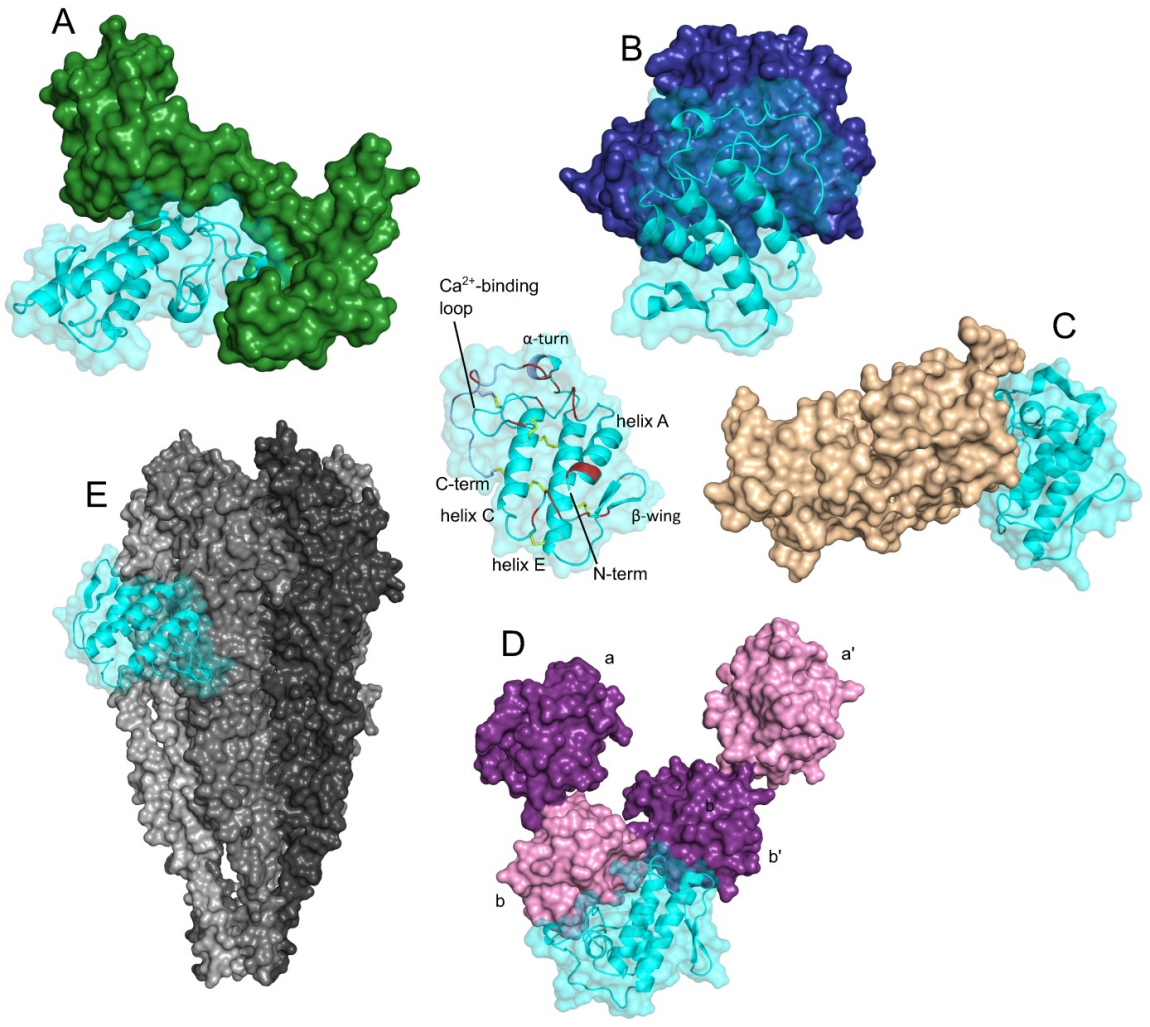
** Secreted PLA_2_s are ligands of proteins that are structurally very diverse.** The three-dimensional models, generated by molecular docking, are showing complexes between the sPLA_2_ Atx and CaM (green, PDB ID: 1CLL) (**A**), Atx and FXa (blue, PDB ID: 2BOH) (**B**), CB_b_ and ΔF508NBD1 of CFTR (PDB ID: 1XMJ) (**C**), Atx and PDI (purple/pink, PDB ID: 4EL1) (**D**), and between the sPLA_2_ vurtoxin and nAChR (grey, PDB ID: 2BG9) (**E**). Centre: The sPLA_2_ Atx, showing its main structural elements. Red, interfacial binding surface; yellow, disulphide bonds; violet, C-terminal region. Note that the sPLA_2_s interact with these different sPLA_2_-BPs in very different ways; i.e., they have multiple protein binding sites, as is characteristic of promiscuous proteins. The Figure was prepared by adaptation of Figures from Kovačič et al. (2010) (**A**), Faure and Saul (2011) (**B**), Faure et al. (2016) (**C**), Oberčkal et al. (2015) (**D**) and Vulfius et al. (2014) (**E**), using PyMOL.

The third type of well-defined structural element present in more than one sPLA_2_-BP is the EF-hand Ca^2+^-binding motif. These sPLA_2_-BPs include CaM, crocalbin and TCBP-49, although the structural aspects of sPLA_2_ binding have been studied only for CaM. A three-dimensional model of a complex between CaM and the snake venom GIIA sPLA_2_ ammodytoxin (Atx) 16 revealed that CaM 'clamps' Atx between its N-terminal and C-terminal domains (Figure 2A). Atx contacts CaM most extensively through a distinct patch of hydrophobic and charged amino acids at its C-terminus, which interact with the central part of CaM, and through the α-helices C and E, which contact the C-terminal globular domain of CaM. In the complex, Atx is oriented in such a way that the entrance to its enzymatic pocket remains open wide. In the complex, a new larger membrane-contacting area (IBS) is formed; this comprises parts of both Atx and CaM, and explains the increased enzymatic activity of complexed Atx.

Considerable structural insight has also been gained for the interactions between the anticoagulant GIIA sPLA_2_s Atx, CB (basic sPLA_2_ subunit of crotoxin, from venom of the South American rattlesnake [*Crotalus durissus terrificus*]) and daboxin P (from venom of the Indian viper [*Daboia r. russelii*]), and human activated blood coagulation factor X (FXa) 40, 56, 57, 58. The interaction area between the anticoagulant sPLA_2_s and FXa is extensive, and encompasses the heavy and light chains of FXa. As evident from the model of the complex between Atx and FXa shown in Figure 2B, Atx interacts with the FXa heavy chain through its Ca^2+^-binding loop, α-helix C, β-wing, and C-terminal region, and with the FXa light chain through its β-wing and α-helices A and B. Electrostatic interactions importantly participate in the efficient binding of sPLA_2_s to FXa, including in particular some positively charged amino acids of Atx (e.g., Arg72, Lys74, His76, Arg77 in the β-wing; Arg118, Lys128, Lys127, Lys132 in the C-terminal region). However, not all of the basic sPLA_2_s are strong anticoagulants, which are consistent with the importance also of certain hydrophobic and aromatic sPLA_2_ amino acids for optimal interactions with FXa (e.g., Atx: Phe124 in the C-terminal region; CB_a2_: Trp70 in the 65-72 loop).

Molecular docking allowed to propose a model of the complex between ΔF508-NBD1 (nucleotide-binding domain 1), the sPLA_2_-binding domain of ΔF508CFTR (Phe508 deletion mutant of cystic fibrosis transmembrane conductance regulator), and CB_b_ (Figure 2C) 33. From the model, which applies also to the wild type NBD1 (i.e. CFTR), it can be seen that the binding interface of CB with ΔF508NBD1/NBD1 is predominately composed of the hydrophobic residues located in its α-helices A and B, Ca^2+^-binding loop and the C-terminal region. Hydrophobic interactions are clearly very important for holding both proteins together, nevertheless, also some polar amino acid residues are involved in the interaction. Forming ionic contacts with ΔF508NBD1/NBD1 of ΔF508CFTR/CFTR, the N-terminal His1 and the Asp112 in the C-terminal region of CB are two examples.

A three-dimensional model of the complex between Atx and PDI has also been reported 20. PDI consists of multiple domains (i.e., a, a', b, b'), and according to the model shown in Figure 2D, the Atx-binding site on PDI is situated between domains b and b'. Atx interacts with PDI across an extensive area that also includes the Atx IBS. The basic amino acids are important for Atx binding to PDI, especially Arg77 and Arg118, but also Lys69, Arg72, Lys74 and Lys86. However, Atx also contacts PDI with some hydrophobic amino acids, Leu3, Leu19 and Phe24, and through two polar amino acids, Asn17 and Asn119.

Molecular docking has also provided some structural insights into the interactions between vurtoxin, another snake venom GIIA sPLA_2_, and the nicotinic acetylcholine receptor (nAChR) 59. According to their model, vurtoxin binds at the interface of the α and γ subunits of nAChR, and is oriented with its active site towards the lipid bilayer (Figure 2E). Vurtoxin does not occupy the nAChR binding sites for its classical agonists and competitive antagonists; however, it is located very close to these.

Several structural features allow the sPLA_2_s to bind to structurally very diverse targets (Table 1; Figure 2), which is characteristic of promiscuous proteins 60, 61. Despite the generally compact structure of sPLA_2_s, the flexibility of the exposed side chains of the amino acids at the IBS promotes their optimal binding to phospholipid aggregates, as their substrates, for efficient hydrolysis 62. As indicated above, the IBS is critically involved also in the interactions of sPLA_2_s with the CRD-containing sPLA_2_-BPs and FXa, as also for PDI 20 and vimentin 26. The association of the sPLA_2_ IBS with a variety of different protein surfaces is most likely driven by the same principles as the association of the sPLA_2_ IBS with phospholipid membranes. The flexibility of the sPLA_2_ β-wing might be another feature that favours their high adaptability for different protein partners. For example, the β-wing of sPLA_2_s should be involved in the interactions of sPLA_2_s with β-PLIs and γ-PLIs 63. sPLA_2_s are also characterized by patches of basic and hydrophobic amino acids that might also greatly broaden the spectrum of their protein interaction partners. We have already shown that such patches are part of the IBS, but they are also located in other parts of sPLA_2_s. Indeed, those at the C-terminus 62 are implicated in the binding of sPLA_2_s to CaM and VEGFR.

However, only the atomic structure of an sPLA_2_-sPLA_2_-BP complex will fully disclose the structural requirements for particular association. The present-day rapid advances in techniques for the determination of the three-dimensional structures of proteins might enable this in the near future; e.g., cryo-electron microscopy.

## Pathophysiological significance of sPLA_2_ binding to sPLA_2_-BPs

**Figure 3:**
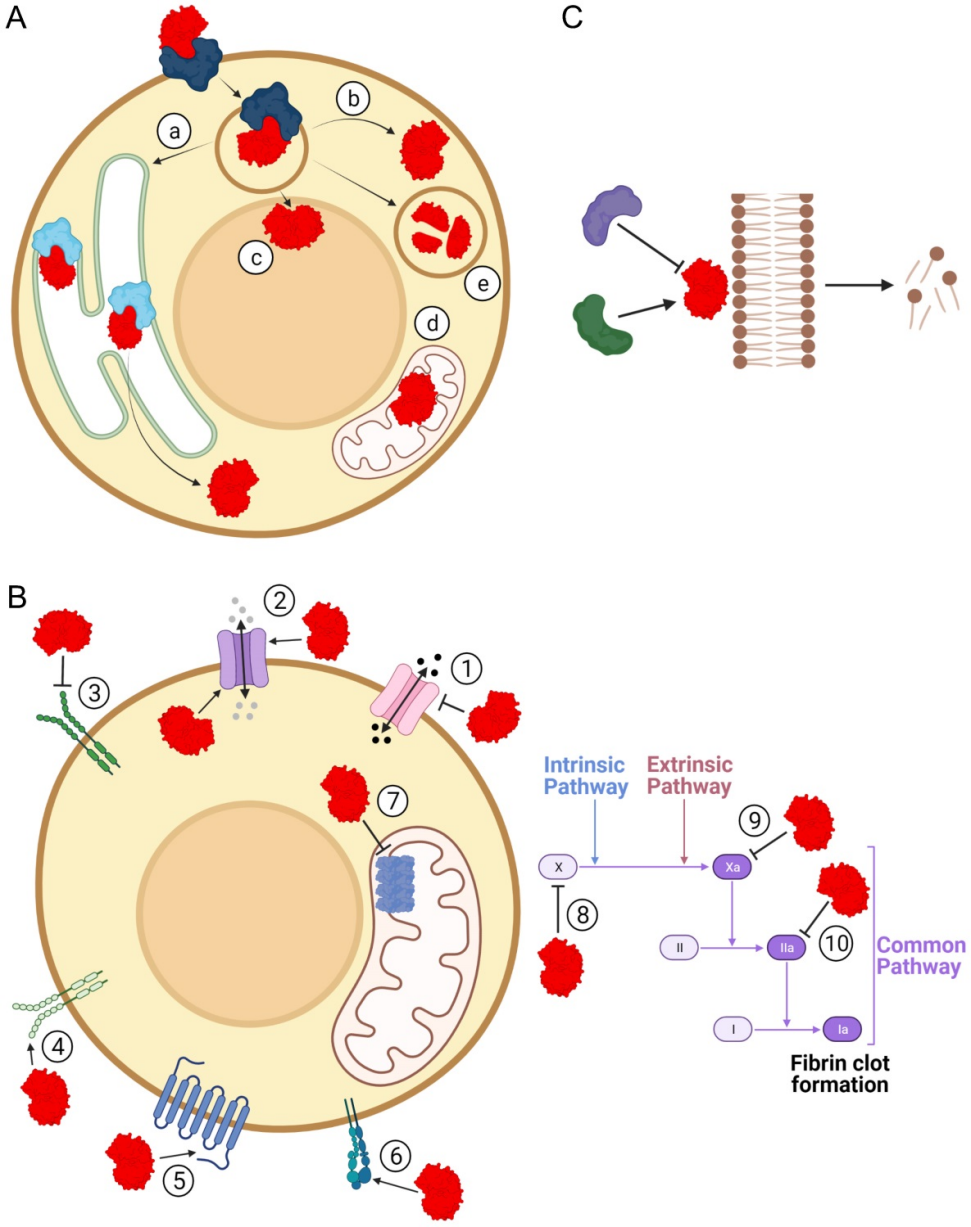
** Pathophysiological implications of binding of sPLA_2_s to sPLA_2_-BPs.** Physiological and/or pathological effects of sPLA_2_s (red) are also the consequence of their binding to sPLA_2_-BPs. (**A**) By binding to sPLA_2_-BPs (blue/cyan), sPLA_2_s can be translocated to specific intracellular compartments, such as the endoplasmic reticulum (a), cytosol (b), nucleus (c), mitochondria (d) or lysosomes (e). In each of these compartments, they can act as enzymes or ligands for receptors, or they can undergo proteolytic degradation in the lysosome. (**B**) As ligands for receptors, sPLA_2_s have been specifically implicated in molecular signalling through decreased (1) or increased (2) permeabilities of certain ion channels, inhibition (3) or activation (4) of activities of receptor tyrosine kinases, modulation of activities of GPCR (5), interference in integrin-mediated functions (6), attenuation of ATP production (7) and inhibition of blood coagulation, at different stages (8, 9, 10). (**C**) Binding of sPLA_2_ to a sPLA_2_-BP might inhibit or potentiate phospholipolytic activity. The Figure was created with BioRender.com.

The structural diversity of sPLA_2_-BPs is consistent with the wide range of pathophysiological activities that have been associated with sPLA_2_s. By binding to sPLA_2_-BPs, sPLA_2_s have been suggested to be involved in inflammation 64, 65, hormone release 66, 67, neurotoxicity 20, 37, 68, 69 and myotoxicity 22. It has already been confirmed that sPLA_2_s are ligands for specific sPLA_2_-BPs in cytokine production 70, cell proliferation 71-73, cell migration 74, lipid mediator production 75, antibacterial activity 73 and blood coagulation 40. These (patho)physiological effects of sPLA_2_s might arise through: (1) specific cellular translocation and associated actions of sPLA_2_s after their binding to sPLA_2_-BPs; (2) triggering of specific signalling following their binding to sPLA_2_-BPs; and/or (3) regulation of sPLA_2_ enzymatic activity by sPLA_2_-BPs (Figure 3). Particular mechanistic possibilities for sPLA_2_ actions as ligands for sPLA_2_-BPs are exemplified and discussed below.

Binding of sPLA_2_s to some sPLA_2_-BPs does not have any obvious physiological function. Some functional implications of sPLA_2_s acting as ligands certainly remain to be discovered, while others might be latent. When environmental conditions change radically, these interactions might become functional. Promiscuous proteins, which include sPLA_2_s, can acquire new functions much more readily than non-promiscuous proteins, which can provide swifter adaptation of the organism to new situations 76, 77.

### Cellular transport of sPLA_2_s after binding to sPLA_2_-BPs

Although sPLA_2_s appear to be secreted into the extracellular space after being synthesized inside the cell, convincing evidence of their intracellular localization and activities has already been provided 69, 71, 78, 79. sPLA_2_s can re-enter cells after their synthesis and secretion, to pass into the cytosol and to different organelles, such as the nucleus and mitochondria 80. Indeed, several sPLA_2_-BPs have been suggested to assist sPLA_2_s in their retrograde cellular transport and intracellular translocation (Figure 3A).

The sPLA_2_-BP in the lumen of the ER, PDI, has been proposed to be involved in such retro-transport of the Atx snake venom GIIA sPLA_2_, which is a neurotoxin from nose-horned viper venom that acts presynaptically 20. Molecular docking and heterologous competition assays have suggested that PDI acts in a similar way as on Atx also on some mammalian sPLA_2_s, such as GIB, GIIA and GV sPLA_2_s.

Two other candidates for the same function are the ER luminal proteins TCBP-49 and crocalbin, which were discovered through their binding to the hetero-oligomeric snake venom sPLA_2_s taipoxin and crotoxin 9. As for PDI, these two EF-hand Ca^2+^-binding proteins have the characteristic ER-retention motif at their C-termini, which has been suggested to be essential for retention and concentration of sPLA_2_s in the ER before they are translocated into the cytosol. Some other EF-hand Ca^2+^-binding proteins might also interact with sPLA_2_s. For instance, the Miro proteins are mitochondrial adaptor proteins that promote the transportation of mitochondria by connecting them to motor proteins 81. Binding of sPLA_2_s to the Miro proteins might concentrate the sPLA_2_s on the outer mitochondrial membrane, which would explain their colocalization with mitochondria after entering cells 78, 79.

Cellular retro-transport of sPLA_2_s might also be associated with two recently discovered sPLA_2_-BPs, vimentin and nucleolin. Vimentin belongs to the family of intermediate filaments, although it is also present on the cell surface and in extracellular fluids. Indeed, in recent years, vimentin has been shown to have a much wider role in cell physiology, rather than just being an inert scaffold protein 82. Vimentin was identified as the receptor for GIIA sPLA_2_ on the surface of apoptotic human T lymphocytes 65. Interactions between these two proteins was also shown in rheumatoid fibroblast-like synoviocytes, which associated rapid internalization of sPLA_2_ with arachidonic acid metabolism in synovial inflammation 83. Recently, vimentin was reported to bind an acidic sPLA_2_ (NnPLA_2_-I) from venom of the Indian cobra (*Naja naja*) 26. Binding of NnPLA_2_-I to vimentin resulted in its internalization into partially differentiated myoblasts. The involvement of vimentin in cellular uptake mechanisms has already been shown for C3, a *Clostridium botulinum* toxin 84, 85, and several viruses, such as human immunodeficiency virus type 1, vaccinia virus and the severe acute respiratory syndrome coronavirus 86. In all of these cases, vimentin acts as a component of the cellular attachment mechanism, either as a receptor or a co-receptor. In a similar way, vimentin might mediate internalization of sPLA_2_s. This is further supported by the similar location of the binding sites on vimentin for dengue virus DENV-2 envelope protein domain III, *Clostridium botulinum* C3 exoenzyme and NnPLA_2_-I, all of which have been proposed to bind to the rod domain of vimentin 26, 84, 87.

Nucleolin was described recently as an sPLA_2_-BP due to its binding to the myotoxin MT-II, a Lys49 sPLA_2_ from venom of a pit viper, the terciopelo (*Bothrops asper*) 27. It appears most likely that nucleolin participates in the toxic mechanism of MT-II through promotion of its translocation from the outside into the perinuclear and nuclear areas of myotubes and macrophages. Nucleolin was previously reported to mediate the internalization of several other molecules from the cell surface to the nucleus through an active Ca^2+^-dependent transport mechanism 88, which might also be the mechanism for sPLA_2_ internalization. It would be interesting to investigate the potential involvement of nucleolin in GV sPLA_2_ translocation to the nucleus, where this sPLA_2_ has been shown to act on the nuclear envelope 89. Nucleolin has been shown to act as a chaperone for histones and TDP-43 90, 91, so it might also stabilize sPLA_2_s in the reducing environment of the cytosol.

Neuronal pentraxins (i.e., NP1, NP2/Narp) are sPLA_2_-BPs that are homologous to acute phase proteins, and these might also function in retrograde transport of sPLA_2_s from the cell surface 28, 92. An sPLA_2_-shuttling function has also been suggested for the ubiquitously expressed eukaryotic cytosolic 14-3-3 proteins, which were discovered through their interactions with Atx 29. The neurotoxicity of Atx also involves its binding to the 14-3-3γ and 14-3-3ε isoforms, to correctly position it at the plasma membrane to hinder the function of amphiphysin, and thus of vesicle endocytosis 69.

HSPGs such as the GPI-anchored glypican I, biglycan, syndecan and perlecan, can bind mammalian sPLA_2_s, e.g. GIIA, GIID and GV sPLA_2_s. Using transfected human embryonic kidney 293 cells, it was shown that a GPI-anchored HSPG facilitated the shuttling of GIIA sPLA_2_ into certain subcellular compartments where the sPLA_2_ then released arachidonic acid for prostaglandin synthesis 93. Binding of human GIIA sPLA_2_ to HSPGs was further demonstrated in human primary T-lymphocytes 25. Recently, it was suggested that GIIA sPLA_2_ can increase endothelial cell permeability after binding to HSPGs on the surface of these cells, in a process that is dependent on the sPLA_2_ phospholipase activity 39. In a similar way, HSPGs might be receptors for heparin-binding snake venom sPLA_2_s. By binding to HSPGs, these toxins might enhance inflammation and permeability of the endothelium, to allow the venom to spread more efficiently in the tissue.

M-type sPLA_2_R can rapidly internalize sPLA_2_s and direct them to the lysosomes, where they are then degraded 94, 95. In this way, M-type sPLA_2_R can down-regulate the activity of sPLA_2_s. M-type sPLA_2_R can also mediate sPLA_2_ signalling by transporting sPLA_2_s into specific intracellular compartments 96, 97. Recent structural studies of the human M-type sPLA_2_R ectodomain using cryo-electron microscopy showed pH-dependent conformational changes that might have important roles in the control of the functional properties of this receptor, including its sPLA_2_ binding 41.

### Signalling triggered by sPLA_2_s as ligands

As proteins that are secreted into the extracellular space, sPLA_2_s encounter various plasma-membrane receptors on target cells. By binding to some of these receptors, sPLA_2_s can induce intracellular responses that are associated with different physiological processes, such as vascular permeability, cell growth, migration and senescence, hormone release, cytokine and NO production, inflammation, cell adhesion and angiogenicity (Figure 3B).

In this context, sPLA_2_s have been reported to interact with different ion channels (K^+^, Na^+^ or Ca^2+^). β-Butx is a neurotoxic sPLA_2_ from venom of the many-banded krait (*Bungarus multicinctus*), and it has been shown to interact with voltage-dependent K^+^ channels. Further, MitTx from venom of the Texas coral snake (*Micrurus t. tener*) was shown to bind to voltage-insensitive Na^+^-conducting acid-sensing ion channels 98. However, both of these toxins are heterodimers that are composed of the sPLA_2_ subunit and a subunit homologous to the Kunitz-type serine protease inhibitor and, with the ion channel binding attributed to the latter. Therefore, these ion channels are not sPLA_2_-BPs. Nonetheless, the neurotoxicity with β-Butx and the pain with MitTx did not occur in the absence of the sPLA_2_ subunit. Some sPLA_2_s can influence the conductance of Ca^2+^ channels, the N-methyl-D-aspartate receptor or L-type voltage-dependent Ca^2+^ channels. Direct binding of sPLA_2_s to these channels has, however, not been demonstrated to date 30, 99.

Recently, pentameric ligand-gated ion channels, nAChRs 31, 59 and their bacterial homologue GLIC from the cyanobacterium *Gloeobacter violaceus* 32, were reported to be sPLA_2_-BPs. By binding to nAChRs, sPLA_2_s might affect nAChR-related functions, such as neuromuscular transmission and cell proliferation. Interestingly, nAChRs have also been described for the outer mitochondrial membrane, and were associated with regulation of the formation of mitochondrial permeability transition pores, which release pro-apoptotic substances like cytochrome c and reactive oxygen species 100. Antagonists of nAChR were shown to attenuate the release of cytochrome c. The antagonistic effect of certain sPLA_2_s on some nAChRs subtypes might therefore link these sPLA_2_s to control of cellular viability through these nAChRs 31, 101.

CFTR is a Cl^-^ channel that was defined as an sPLA_2_-BP using CB, the basic sPLA_2_ subunit of crotoxin 33. CB was shown to bind and allosterically potentiate the activity of CFTR. Of a very high medical significance, CB was found to bind with nanomolar affinity also to ΔF508CFTR mutant, the causative factor of cystic fibrosis, thus augmenting its activity. Importantly, by binding to ΔF508CFTR, CB acts also as a corrector, facilitating trafficking and delivery of the abnormal protein to the plasma membrane.

VEGFRs are receptor tyrosine kinases that can interact with some myotoxic snake venom sPLA_2_s. Although some of these toxins are potent antagonists of VEGFRs, it is still not clear whether their binding to VEGFRs is directly involved in their sPLA_2_ myotoxicity 22, 23. Nevertheless, the interactions between snake venom sPLA_2_s and VEGFRs might be important to enhance vascular permeability, to facilitate penetration of the snake venoms into tissues.

Epidermal growth factor receptor (EGFR) is another receptor tyrosine kinase that interacts with sPLA_2_s. It was reported to interact with human GIIA sPLA_2_ 34. However, in this case, the sPLA_2_ acted as an agonist, as it up-regulated HER (human EGFR)/HER2-elicited signalling in lung cancer, thus stimulating cancer cell growth.

M-type sPLA_2_R has already been mentioned as an sPLA_2_-translocator (see section 4), and it can also act as a signalling receptor to transduce sPLA_2_-dependent signals independent of sPLA_2_ catalytic activity. Through M-type sPLA_2_R, sPLA_2_s have been implicated in cell proliferation, migration and senescence, and in hormone release and cytokine and NO production. It was also suggested that human GIB sPLA_2_ can induce kidney glomerular podocyte apoptosis via M-type sPLA_2_R 102.

PA2-Vb is an acidic sPLA_2_ from venom of the Chinese green tree viper (*Trimeresurus stejnegeri*), and it was shown to induce mouse aorta contraction independent of its enzymatic activity, by acting on a protease-activated receptor (PAR-1), which is a GPCR 35. GPCRs are the largest and most diverse class of membrane receptors in eukaryotes, and their primary function is to transduce extracellular stimuli into intracellular signals, which lead to different cell responses.

Integrins are extracellular plasma membrane (transmembrane) proteins that are responsible for cell adhesion to the extracellular matrix. They are dimers, as combinations of one of 18 α-subunits and one of 8 β-subunits. Integrins α_v_β_3_ and α_4_β_1_ have been shown to bind mammalian GIIA sPLA_2_, while an acidic GIIA sPLA_2_ from venom of the viper *Macrovipera lebetina transmediterranea* was shown to interact with integrins α_5_β_1_, α_v_β_3_ and α_v_β_6_, to induce inflammation and inhibition of cell adhesion and migration and angiogenicity 36, 103.

CCOX is an essential constituent of the respiratory chain complex, and was characterized as an sPLA_2_-BP due to its binding to Atx 37. Through binding to subunit II, Atx was shown to inhibit the oxidase activity of CCOX, independent of its phospholipase activity. This might explain the inhibition of ATP production in nerve endings poisoned by the snake venom neurotoxic sPLA_2_s 104. These findings also provide novel indications for the potential functions and malfunctions of the orthologous mammalian GIIA sPLA_2_ in mitochondria.

The blood coagulation system consists of a strictly regulated proteolytic cascade of blood coagulation factors that convey signals downstream to fibrinogen, which is then transformed into the insoluble fibrin network, and to activate platelets. Ultimately, a blood clot is formed to prevent blood loss from the injured blood vessel 105. Several sPLA_2_s can affect this process by binding to one of these coagulation factors, to thus inhibit their functions (Figure 3B). Some snake venom sPLA_2_s bind to FX, or to its activated form FXa, or to thrombin (FIIa); e.g., crotoxin and Atx can induce anticoagulant effects through binding to FXa, to thus prevent formation of the prothrombinase complex 40, 106. Human GIIA sPLA_2_ acts on blood coagulation in exactly the same way 107. Daboxin is an anticoagulant sPLA_2_ that can bind to both FXa and FX 57. On the other hand, Nk-PLA_2_α and Nk-PLA_2_β from venom of the monocled cobra (*Naja kaouthia*) act as anticoagulants through their binding to thrombin and their consequent inhibition of its proteolytic activity 108.

### Regulation of sPLA_2_ enzymatic activity by sPLA_2_-BPs

sPLA_2_s participate in many important physiological processes through their enzymatic activity 2. These range from those dependent on the composition and flexibility of biological membranes, to those regulated by the products of the sPLA_2_ phospholipolytic activity, which include lysophospholipids, free fatty acids and their metabolites - the whole range of signalling hormones 109,110. Regulation of the enzymatic activity of sPLA_2_s is thus extremely important for the correct functioning of an organism, and this regulation is also mediated through the binding of sPLA_2_s to particular sPLA_2_-BPs (Figure 3C).

M-type sPLA_2_R has been shown to regulate the activity of sPLA_2_s in two ways: through acting as an sPLA_2_ inhibitor; and through mediating sPLA_2_ endocytosis, which leads to sPLA_2_ degradation in lysosomes 111. Soluble form of M-type sPLA_2_R that is detected in the blood can function only in the first way 8. Pulmonary SP-A is structurally and functionally similar to the soluble form of M-type sPLA_2_R. SP-A belongs to the CRD-containing family of proteins, and it inhibits the enzymatic activity of GIIA and GX sPLA_2_s in pulmonary surfactant 9.

Soluble proteins that inhibit sPLA_2_s have been isolated from the blood of snakes and some other animals. These are ecologically connected with venomous snakes, and are known as the α-PLIs, β-PLIs and γ-PLIs 9.

sPLA_2_ inhibitors have also been found in plants. *Withania somnifera* glycoprotein (WSG) is an acidic glycoprotein that is similar to the α-chain of the γ-PLIs, and it was isolated from the medicinal plant known variously as Ashwagandha, Indian ginseng and winter cherry. WSG inhibits the enzymatic activity and toxicity of NN-XIa-PLA_2_, an sPLA_2_ myotoxin from venom of the Indian cobra 112-114.

Interestingly, sPLA_2_-BPs have also been shown to increase the enzymatic activity of sPLA_2_s. One such sPLA_2_-BP is the EF-hand Ca^2+^-binding intracellular protein CaM. When sPLA_2_s are in a complex with CaM, they also become more resistant to chemical denaturation 16, 17. Two other sPLA_2_-BPs that can potentiate the catalytic activity of sPLA_2_s are vimentin 65 and GLIC 32. The potentiating effect appears to occur because the interactions with these three sPLA_2_-BPs position the complexed sPLA_2_ on the membrane in a way that provides more efficient catalytic function.

## Medical potential of sPLA_2_s as ligands for receptors

sPLA_2_-BPs have diverse physiological functions, and therefore their interactions with sPLA_2_s can be insightful and potentially helpful for medical applications. Important roles of M-type sPLA_2_R in cancers have been outlined recently, with a tumour suppressive role demonstrated 74, 115. As ligands of M-type sPLA_2_R, sPLA_2_s can be used to study or to regulate processes in which M-type sPLA_2_R is involved. By binding to M-type sPLA_2_R, sPLA_2_s have already been indicated to have roles in cell proliferation, migration and senescence, hormone release, and cytokine and NO production. sPLA_2_s were also demonstrated to induce kidney glomerular podocyte apoptosis via M-type sPLA_2_R 102. A recently generated conditional transgenic mouse that expresses human M-type sPLA_2_R1 will certainly facilitate future investigations into this sPLA_2_-BP under different pathophysiological conditions 116.

VEGFRs are sPLA_2_-BPs that have also been shown to be involved in cancer development. By binding to VEGFRs, sPLA_2_s might inhibit angiogenesis, which is an essential process for cancer metastasis formation 117. Therefore, the use of sPLA_2_s in chemotherapy has been proposed. In addition, the angiogenic pathways of VEGFRs and endothelial nAChRs have been shown to have cross-talk, which suggests that as antagonists of nAChRs, sPLA_2_s might further inhibit angiogenesis in this way 31, 118. In contrast to the antagonistic effects on VEGFRs and nAChRs, GIIA sPLA_2_ can activate EGFRs in lung cancer cells 34. This leads to elevated HER /HER2-elicited signalling, which contributes to overexpression of GIIA sPLA_2_. Plasma concentration of GIIA sPLA_2_ might therefore serve as a biomarker for lung cancer, and GIIA sPLA_2_ might represent a therapeutic target to treat patients with lung cancer.

The integrin binding of snake venom sPLA_2_s has also been exploited for development of new anti-cancer agents that target cell proliferation and migration 36, 103. MT-II binding to nucleolin appears to explain the higher toxicity of MT-II against cancer cells, as nucleolin is more abundant on the surface of cancer cells than of normal cells 90. Nucleolin also participates in internalization of many viruses, which suggested that the anti-viral activity of some sPLA_2_s might be due to their nucleolin binding 27.

The involvement of GIIA sPLA_2_ in synovial inflammation through liberation of arachidonic acid for production of inflammatory eicosanoids has long been known. Efforts have been made to develop inhibitors of GIIA sPLA_2_ to attenuate rheumatoid arthritis and sepsis, but without substantial success so far 119, 120. Colocalization of GIIA sPLA_2_ and vimentin is, however, associated with phospholipase-activity-independent mechanisms of signalling through arachidonic acid metabolism 83, which provides the way for targeted studies of GIIA sPLA_2_ signalling, and for the development of new therapeutic strategies based on inhibition of GIIA sPLA_2_. Lee et al. (2013) 83 also identified vimentin as an interesting player in some other diseases where the involvement of GIIA sPLA_2_ has been indicated, and particularly in cancers. GIIA sPLA_2_ is overexpressed in many types of cancers, while vimentin is one of the signature biomarkers of tumour dedifferentiation through epithelial-mesenchymal transition 121, 122.

Abundant expression of vimentin has also been observed in adult neurons as a response to injury, such as in Alzheimer's disease 123. As vimentin might assist the internalization of GIIA sPLA_2_ into neurons, this suggests why GIIA sPLA_2_ is involved in the aetiology of Alzheimer's disease. GIIA sPLA_2_ might directly damage neurons or boost inflammation by releasing excessive arachidonic acid. Targeting of vimentin is, therefore, potentially interesting as a new therapeutic approach to treat patients with Alzheimer's disease.

A phospholipase-activity-independent mode of action is also characteristic for snake venom catalytically inactive sPLA_2_s. These can induce inflammation 124; e.g., MT-II can stimulate the production and release of inflammatory mediators, such as interleukin-6 125, interleukin-1, tumour necrosis factor α, leukotriene B4, thromboxane A2 and prostaglandins E2 and D2 126-129. Indeed, this activity of MT-II was recently used to establish a new experimental model of acute arthritis 130.

Mammalian GIB, GIIA, GV and GX sPLA_2_s induce the production of pro-inflammatory cytokines and chemokines, whereby these sPLA_2_s increase inflammation after binding to HSPGs or to M-type sPLA_2_R on macrophages, neutrophils, eosinophils, monocytes and endothelial cells 131. This binding to HSPGs also participates in clearance of these sPLA_2_s and in reduction of their enzymatic activity towards low-density lipoprotein, which is an important factor in atherosclerosis 3, 132.

sPLA_2_s also mediate pro-inflammatory actions via integrins, as binding of GIIA sPLA_2_ to human integrins α_v_β_3_, α_4_β_1_ and α_5_β_1_ trigger signalling that leads to inflammation 36, 133. The effects of sPLA_2_s on haemostasis have great potential for medical applications as well. The induction of vasoconstriction by PA2-Vb binding to PAR-1 makes PA2-Vb interesting for the development of new therapeutic approaches against hypertension, atherosclerosis and diabetes-related vascular problems 35. Moreover, snake venom sPLA_2_s can induce strong anticoagulant effects through competitive binding to constituents of the prothrombinase complex, which suggests their great potential for the development of therapeutic procedures to attenuate or prevent blood-clot formation 108.

As already mentioned, GIIA sPLA_2_ has been associated with the aetiology of some neurodegenerative diseases, such as Alzheimer's disease 134-137. A hallmark of the induction of Alzheimer's disease is the elevated expression of GIIA sPLA_2_ in the affected tissue, with concomitant dysfunction of the neuronal mitochondria. As the pathological effects of presynaptically neurotoxic sPLA_2_s from snake venoms and GIIA sPLA_2_ on mitochondria are similar, a description of the mode by which neurotoxic sPLA_2_s encounter and affect neuronal mitochondria at the molecular level is expected to advance the study of the role of endogenous GIIA sPLA_2_ in this and other related destructive diseases.

These findings indicate a way to the development of original diagnostic and therapeutic solutions. An important breakthrough in this direction was the recent identification of CCOX as the mitochondrial receptor for Atx 37. Application-wise, a very promising discovery was also the binding of crotoxin to ΔF508CFTR, responsible for cystic fibrosis. The sPLA_2_ subunit of crotoxin has been used as a template to develop a new line of anti-cystic fibrosis agents 33.

## Conclusions and outlook

As well as participating in a very broad spectrum of biological processes through their enzymatic activity, sPLA_2_s participate in many physiological settings as ligands for membrane or soluble receptors. As new and more sensitive analytical tools are developed, the number of newly discovered sPLA_2_-BPs is growing. Further technical advances will promote the discovery of even more sPLA_2_-BPs.

sPLA_2_-BPs are structurally diverse integral membrane and soluble proteins. Despite many attempts, their common sPLA_2_-binding attributes remain largely obscure. The solution to this puzzle will enable targeted searches of additional sPLA_2_-BPs. A straightforward approach to solve this problem would be the determination of the three-dimensional structures of complexes between sPLA_2_s and their sPLA_2_-BPs. With the development of powerful new technologies, such as cryo-electron microscopy, this is becoming more and more realistic.

sPLA_2_s are very useful molecules from the evolutionary point of view. Particular structural features have made the sPLA_2_s promiscuous, and promiscuous proteins can acquire new functions more readily than other proteins. In this way, these can enable organisms to adapt more successfully to environmental changes.

Finally, due to their enzymatic activity and extensive interactomes, sPLA_2_s are exceptional pharmacological targets. Detailed descriptions of their actions at the molecular level will initiate the development of a plethora of original diagnostic and therapeutic approaches.

## Figures and Tables

**Table 1:** Some characteristics of sPLA_2_-BPs.

sPLA_2_-BP	Location	Defined structural element	Interacting part of sPLA_2_	Binding affinity	Effect of interaction
M-type sPLA_2_Rs 10	Plasma membrane, extracellular	CRD-like fold	Ca^2+^-binding loop	38 pM (OS_1_) 11	Inhibition, clearance, translocation of sPLA_2_
α-PLIs 12	Blood		n.d.	n.d.	Inhibition of sPLA_2_
SP-A 13	Extracellular		n.d.	n.d.	Inhibition of sPLA_2_
β-PLIs 14	Blood	Leucine-rich repeat	N-terminal region, β-wing	n.d.	Inhibition of sPLA_2_
γ-PLIs 15	Blood	Three-finger motif	N-terminal region, β-wing	n.d.	Inhibition of sPLA_2_
CaM 16	Cytosol	EF-hand Ca^2+^-binding motif	C-terminal region, α-helices C, E	3.3 nM (Atx) 17	Stabilization of sPLA_2_, augmentation of sPLA_2_ enzymatic activity
TCBP-49 18	Endoplasmic reticulum		n.d.	n.d.	Translocation of sPLA_2_ (proposed)
Crocalbin 19	Endoplasmic reticulum		n.d.	n.d.	Translocation of sPLA_2_ (proposed)
PDI 20	Endoplasmic reticulum	Thioredoxin-like fold	IBS	1.27 µM (Atx) 21	Translocation of sPLA_2_ (proposed)
VEGFR-1/Flt-1 22	Plasma membrane	Ig-like domain	C-terminal region	74 nM (Lys49 GIIA) 23	Competitive inhibition of VEGFR
VEGFR-2/KDR 22	Plasma membrane			10 nM (Lys49 GIIA) 23	
DM64 24	Blood		n.d.	n.d.	Neutralization of sPLA_2_
HSPGs 25	Plasma membrane	Negatively charged carbohydrate moiety	Clusters of basic amino acids at C- and N-terminal regions	n.d.	Clearance and translocation of sPLA_2_
Vimentin 26	Cytosol, plasma membrane	Rod domain	IBS	n.d.	Internalization and translocation of sPLA_2_
Nucleolin 27	Nucleolus, cytoplasm, plasma membrane	RNA recognition motif	n.d.	n.d.	Internalization and translocation of sPLA_2_
NP1, NP2, NPR 28	Extracellular	Pentraxin domain	n.d.	n.d.	Translocation of sPLA_2_ (proposed)
14-3-3γ/ε 29	Cytosol	14-3-3 domain	C-terminal region	1 µM (Atx) 29	Positioning of sPLA_2_ on plasma membrane
L-type voltage-dependent Ca^2+^ channel 30	Plasma membrane	α domain	n.d.	n.d.	Activation of L-type voltage-dependent Ca^2+^ channel (proposed)
nAChR 31	Plasma membrane	-	n.d.	120 nM (crotoxin) 31	Negative allosteric modulation of nAChR
GLIC 32	Plasma membrane	ECD	IBS	125 nM (CB_c_) 32	Negative allosteric modulation of GLIC
CFTR/ ΔF508CFTR 33	Plasma membrane	NBD1	IBS, Ca^2+^-binding loop, C-terminal region	4 nM (CB_c_) 33	Potentiation and correction of ΔF508CFTR
EGFR 34	Plasma membrane	L domain	n.d.	n.d.	Activation of EGFR
PAR-1 35	Plasma membrane	-	n.d.	n.d.	Activation of PAR-1
Integrins 36	Plasma membrane	-	C-terminal region, α-helices D, E	200 nM (hGIIA) 36	Induction of integrin-mediated signalling
CCOX-II 37	Inner mitochondrial membrane	-	C-terminal region	15 nM (Atx) 38	Inhibition of CCOX (proposed)
C1 inhibitor protein 39	Extracellular	-	n.d.	n.d.	Impairment of C1 inhibitor protein activity (proposed)
FX, FXa, FIIa (thrombin) 40	Blood	EGF-like domain of light chain, interface regions I-V, exosite of heavy chain	Basic amino acids in C-terminal region, IBS, loop preceding β-wing	0.6 nM (CB_c_) 40	Noncompetitive inhibition of FX, FXa, FIIa (thrombin)

sPLA_2_-BP, sPLA_2_-binding protein; M-type, muscle-type; CRD, carbohydrate recognition domain; OS_1_, sPLA_2_ from *Oxyuranus s. scutellatus* venom; PLIs, sPLA_2_ inhibitors; n.d., not defined; SP-A, pulmonary surfactant protein A; CaM, calmodulin; Atx, sPLA_2_ from *Vipera a. ammodytes* venom; TCBP-49, taipoxin-associated 49-kDa Ca^2+^-binding protein; PDI, protein disulphide isomerase; VEGFR, vascular endothelial growth factor receptor; DM64, metalloproteinase inhibitor from *Didelphis marsupialis* blood; HSPGs, heparan sulphate proteoglycans; IBS, interfacial binding surface; NP, neuronal pentraxin; nAChR, nicotinic acetylcholine receptor; GLIC, proton-gated ion channel from *Gloeobacter violaceus*; ECD, extracellular domain; CB, basic sPLA_2_ subunit of crotoxin; CFTR, cystic fibrosis transmembrane conductance regulator; NBD; nucleotide-binding domain; EGF(R), epidermal growth factor (receptor); PAR, protease-activated receptor; hGIIA; human group IIA sPLA_2_; CCOX, cytochrome c oxidase; FX/FXa/FIIa, blood coagulation factors.

## Abbreviations

Atx: sPLA_2_ from *Vipera a. ammodytes* venom
CaM: calmodulin
CB: basic sPLA_2_ subunit of crotoxin
CCOX: cytochrome c oxidase
CFTR: cystic fibrosis transmembrane conductance regulator
CRD: carbohydrate recognition domain
DM64: metalloproteinase inhibitor from *Didelphis marsupialis* blood
ECD: extracellular domain
EGF(R): epidermal growth factor (receptor)
ER: endoplasmic reticulum
FX/FXa/FIIa: blood coagulation factors
GLIC: proton-gated ion channel from *Gloeobacter violaceus*
GPCR: G-protein-coupled receptor
HER: human EGFR
hGIIA: human group IIA sPLA_2_
HSPGs: heparan sulphate proteoglycans
IBS: interfacial binding surface
Ig-like: immunoglobulin-like
M-type sPLA_2_Rs: muscle-type sPLA_2_ receptors
nAChR: nicotinic acetylcholine receptor
NBD: nucleotide-binding domain
NP: neuronal pentraxin
OS_1_: sPLA_2_ from *Oxyuranus s. scutellatus* venom
PAR: protease-activated receptor
PDI: protein disulphide isomerase
PLIs: sPLA_2_ inhibitors
SP-A: pulmonary surfactant protein A
sPLA_2_: secreted phospholipase A_2_
sPLA_2_-BP: sPLA_2_-binding protein
TCBP-49: taipoxin-associated 49-kDa Ca^2+^-binding protein
VEGFR: vascular endothelial growth factor receptor
WSG: *Withania somnifera* glycoprotein
